# Gene Expression Analysis of Mevalonate Kinase Deficiency Affected Children Identifies Molecular Signatures Related to Hematopoiesis

**DOI:** 10.3390/ijerph18031170

**Published:** 2021-01-28

**Authors:** Simona Pisanti, Marianna Citro, Mario Abate, Mariella Caputo, Rosanna Martinelli

**Affiliations:** Department of Medicine, Surgery and Dentistry ‘Scuola Medica Salernitana’, University of Salerno, Via Salvatore Allende, 84081 Baronissi (SA), Italy; mcitro@unisa.it (M.C.); mabate@unisa.it (M.A.); macaputo@unisa.it (M.C.)

**Keywords:** mevalonate kinase deficiency, anemia, hematopoiesis, stress erythropoiesis

## Abstract

Mevalonate kinase deficiency (MKD) is a rare autoinflammatory genetic disorder characterized by recurrent fever attacks and systemic inflammation with potentially severe complications. Although it is recognized that the lack of protein prenylation consequent to mevalonate pathway blockade drives IL1β hypersecretion, and hence autoinflammation, MKD pathogenesis and the molecular mechanisms underlaying most of its clinical manifestations are still largely unknown. In this study, we performed a comprehensive bioinformatic analysis of a microarray dataset of MKD patients, using gene ontology and Ingenuity Pathway Analysis (IPA) tools, in order to identify the most significant differentially expressed genes and infer their predicted relationships into biological processes, pathways, and networks. We found that hematopoiesis linked biological functions and pathways are predominant in the gene ontology of differentially expressed genes in MKD, in line with the observed clinical feature of anemia. We also provided novel information about the molecular mechanisms at the basis of the hematological abnormalities observed, that are linked to the chronic inflammation and to defective prenylation. Considering the broad and unspecific spectrum of MKD clinical manifestations and the difficulty in its diagnosis, a better understanding of MKD molecular bases could be translated to the clinical level to facilitate diagnosis, and improve management and therapy.

## 1. Introduction

Mevalonate kinase deficiency (MKD) is a rare disorder of childhood with autosomal recessive transmission that is part of the group of systemic auto-inflammatory diseases called “periodic fevers”, characterized by the presence of continuous, periodic, and recurrent febrile attacks [1]. It is caused by an enzymatic deficiency of the mevalonate kinase enzyme (MVK) due to several possible mutations in the *Mvk* gene. MKD presents as a continuous spectrum of phenotypic manifestations with increasing severity, from the less severe form, known as type D hyperimmunoglobulinemia (HIDS), to the much more disabling form, mevalonic aciduria (MA). The severity of the disease is strictly dependent on the extent of the enzymatic defect. Indeed, in HIDS, the residual activity of MVK is greater in percentage (between 1.8 and 28%) than that in MA, where it is close to zero (<0.5%). To date, a total of 227 sequence variants for the *Mvk* gene have been described. Most of the mutations compromise the stability of the MVK enzyme, but not its catalytic activity, justifying the possible impact that other factors may have on disease expression [2]. In HIDS, heterozygous mutations affect the protein throughout, as to MA, where mutations, mainly homozygous, are usually grouped in the same region, in the core of the protein [3]. MVK is present in all cells of the body and plays a fundamental role in the metabolic pathway of mevalonate, as it is responsible for the synthesis of cholesterol and isoprenoid molecules, which are involved in fundamental cellular processes, including inflammation. Although the precise pathogenesis of MKD is still unclear, the collected evidence suggests that protein prenylation deficiency, due to the lack of isoprenoid moieties, leads to activation of the innate immune response as well as systemic inflammation with hypersecretion of the inflammatory cytokine IL1β [4,5].

From an epidemiological point of view, according to data obtained from the “Eurofever” registry, it is estimated that MKD affects at least 300 people worldwide, with a majority representing the HIDS phenotype rather than MA, in turn even rarer (10%) [6]. Some clinical features are in common between HIDS and MA pathological forms, such the onset of symptoms usually before two years of life, high fever lasting about 3–7 days with chills and headache, presence of mevalonic acid in the urine, persistent elevated levels of IgA, IgE, and in particular, IgD immunoglobulins, and typical systemic inflammatory reactions with increased inflammation indices during the attacks (Erythrocyte sedimentation rate -ESR-, C-reactive protein -CRP-, leukocytosis). Various and widespread pains, hepatosplenomegaly, lymphadenopathy, abdominal symptoms (vomiting and diarrhea), arthralgia and skin rashes are present in both phenotypes. Other common characteristics are the possible triggers of the syndromes, such as vaccinations, viral infections, situations of physical and emotional stress (including trauma and surgery) and the fact that there are no precise pathognomonic symptoms. The prognosis for MA is severe, with mortality >50% in the infancy due to multiorgan failure during inflammatory attacks. Moreover, MA is usually dominated by significant neurological involvement, characterized by alterations such as psychomotor and growth retardation, lack of coordination of movements, progressive cerebellar ataxia, seizures. In addition, facial dysmorphism (frontal bossing, hypertelorism, triangle facies) and progressive visual impairment related to uveitis, cataract, and tapeto-retinal degeneration may be found. Increases in creatine kinase (CK) and transaminases are present in the most severely affected subjects. Hematologic abnormalities with normocytic hypoplastic anemia, leukocytosis, thrombocytopenia, and abnormal forms of blood cells, sometimes predominate in MKD and may lead to initial misdiagnoses of congenital infection or myelodysplastic syndromes. Several patients require blood transfusion due to low hemoglobin levels [7,8]. Some patients that have been treated with hematopoietic stem cell transplantation, in order to reverse the autoinflammatory manifestations through the hematopoietic system replacement, reported a general improvement [9,10,11,12,13]. Such treatment was carried out in very severe cases or when the pharmacological treatment was ineffective. To date, the election therapy is carried out with biological agents targeting interleukin 1 beta (IL1β), anakinra (recombinant human IL1 receptor a, rhIL1Ra) that acts as a competitive inhibitor of IL1, and canakinumab (humanized IgG1 antibody) that binds and neutralizes IL1β. Etanercept, a dimeric tumor necrosis factor (TNF) receptor p75-Fc fusion protein that inhibits TNF-α and tocilizumab, a monoclonal antibody that inhibits the binding of IL6 to its receptor, have been used in patients that do not respond to the first-choice therapy. Non-steroidal anti-inflammatory drugs (NSAIDs) and glucocorticoids, alone or in combination, are administered during the febrile attacks to relieve acute and painful symptoms [13,14].

Toward a better understanding of the pathogenesis of MKD at the molecular level, in this work we performed a bioinformatic analysis to identify genes differentially expressed in peripheral blood cells in MKD patients relative to healthy controls. Through this analysis we found that genes differentially expressed in MKD patients are elements of pathways regulating key biological functions, physiologically related to the symptoms of the disease, in particular hematopoiesis and anemia, a clinical feature observed in almost all patients. We also provided some cues about the molecular mechanisms at the basis of the hematological alterations observed that are strictly dependent on the inflammatory process and on the defective prenylation of key proteins in the context of MKD.

## 2. Materials and Methods

### 2.1. Microarray Data Information and Identification of Differentially Expressed Genes

We performed our analysis using gene expression data of peripheral blood mononuclear cells (PBMC) from 8 MKD children with active disease and 14 healthy children as controls, available in the dataset GSE43553 (https://www.ncbi.nlm.nih.gov/geo/). Clinical data for these patients have been already described elsewhere [15]. The microarray platform utilized for the experiment was [HG-U133A_2] Affymetrix Human Genome U133A 2.0 Array. Differentially expressed genes were identified using GEO2R (https://www.ncbi.nlm.nih.gov/geo/geo2r/). GEO2R is a free available web tool based on R-package which allows us to compare two or more groups of samples from GEO datasets and extract common differentially expressed genes ranked by *p*-value corrected using the Benjamini and Hochberg algorithm (false discovery rate, FDR). Value data were checked to be median centered across samples. We set *p*-value <0.05 and log_2_ fold change (FC) ≥1.4 and ≤−1.4 as the cut-off criteria. The average log_2_ FC was reported for genes identified by more than one probe.

### 2.2. Gene Ontology Enrichment Analysis

Enriched biological functions associated with differentially expressed genes were identified using the Cytoscape (v 2.5.7) plugin ClueGO/CluePedia (v 1.5.7) [16,17]. The integrated databases CLINVAR Human diseases and GO Biological process EBI Uniprot GOA were used as ontologies. The selection criteria included a minimum of 3 genes in the cluster with GO tree interval range in between 3 and 8 and a kappa score of 0.4 for network connectivity. Statistical assessment was conducted with a two-sided hypergeometric test and Benjamini–Hochberg correction. We set *p*-value < 0.001 as the cut-off criterium.

### 2.3. Ingenuity Pathway Analysis

Functional and network analyses of statistically significant gene expression changes were performed through Ingenuity Pathway Analysis (IPA) software (Qiagen, CA, USA, https://www.qiagenbioinformatics.com/products/ingenuitypathway-analysis). In brief, data sets with gene identifiers and fold change values calculated from the microarray experiment were analyzed by IPA. The “core analysis” function was used to interpret the data, through the analysis of biological processes, canonical pathways, upstream transcriptional regulators, and regulatory networks enriched with differentially regulated genes. *p*-values were calculated using a right-tailed Fisher’s exact test. A z score <−2 and >2, which takes into account the directionality of the effect observed, was considered statistically significant. To obtain the functional networks, the differentially expressed genes from the dataset were superimposed on a global meshwork from existing information in the manually curated Ingenuity’s Knowledge Base. Graphical representation of the networks was algorithmically generated based on the connectivity between proteins, represented as nodes connected by a line which indicates the type of biological relationship. Nodes’ color intensity is related to the degree of up- (red) or down- (green) regulation.

## 3. Results

### 3.1. Differentially Expressed Genes in MKD Patients

We compared the expression profiles of PBMC from MKD patients (*n* = 8) with control subjects (*n* = 14). The microarray dataset represents 14,500 different human genes. A total of 258 transcripts, corresponding to 226 genes, showed a significant differential expression in MKD patients vs. control subjects, calculated using the Benjamini and Hochberg procedure (FDR adjusted *p*-value < 0.05 and −1.4 ≤ FC ≥ 1.4) (Supplementary Table S1). The top 25 differentially expressed genes, ordered by FC, are listed in Table 1 (upregulated genes) and Table 2 (downregulated genes).

### 3.2. Gene Ontology Enrichment of Biological Processes and Functional Analysis

**Figure 1:**
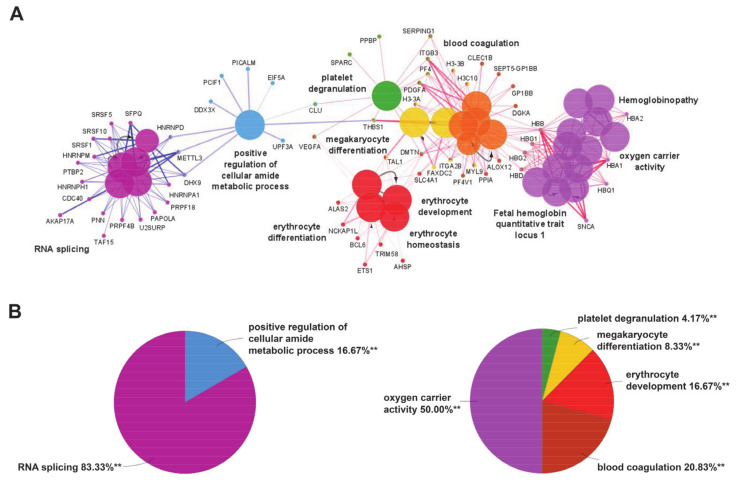
(**A**) Enrichment by gene ontology terms was performed using the ClueGO/CluePedia plugin (v 1.5.7) in Cytoscape software (v 2.5.7). The connectivity of the GO terms network is illustrated by functional nodes and edges that are shared between the differentially expressed genes (downregulated genes in blue and upregulated genes in red) with a kappa score threshold of 0.4. The enrichment shows only highly significant GO terms (*p*-value ≤ 0.001). The node size shows the term significance. The node color indicates the specific functional annotation of ontologies in which the genes are involved. Functionally related groups partially overlap. The most significant terms of a group are highlighted in the network in bold fonts. The names of the differentially expressed genes involved in each group are displayed in uppercase font. (**B**) Pie charts of the enriched biological functions illustrating the %terms/group based on gene ontology classification of the differentially expressed genes (downregulated on the left and upregulated on the right) (** *p*-value ≤ 0.001).

Gene ontology enrichment analysis was performed by ClueGO and mapped for biological processes and human diseases through the integrated databases GO Biological process and CLINVAR (to evaluate the correlation of MKD with other pathologies) for the two separate clusters of downregulated and upregulated genes in MKD. In order to focus our attention only on the most significant processes, we set a very stringent *p*-value (<0.001) in our analysis. We found highly significant overrepresentation of a number of enriched biological functions both for downregulated genes (Figure 1A, blue edges), mostly related to RNA splicing (Figure 1B, pie chart on the left) and upregulated ones (Figure 1B, red edges), which include oxygen carrier activity, blood coagulation, and erythrocyte development (Figure 1B, pie chart on the right). The diseases annotations highlighted the correlation of MKD with several forms of anemia, like hemoglobinopathies and fetal hemoglobin quantitative trait locus 1 (Figure 1A).

### 3.3. IPA Ingenuity Pathway Analysis

**Figure 2:**
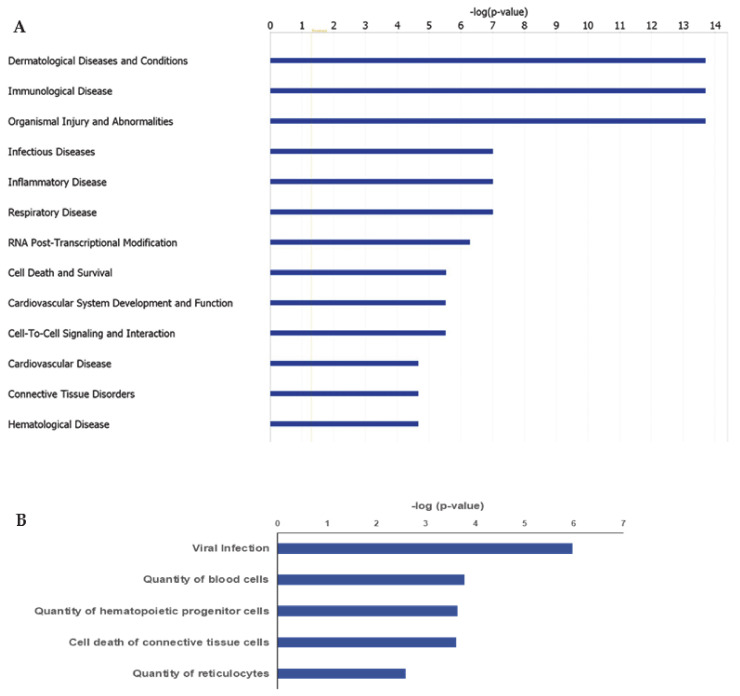
Ingenuity Pathway Analysis (IPA): Illustrated are the top-scoring diseases (**A**) and biological functions (**B**) based on differentially expressed genes in MKD patients. Blue bars in (**B**) refer to a negative z score.

IPA ingenuity pathway analysis was used to predict biological functional processes associated to differentially expressed genes in MKD. Diseases and disorders predicted to be influenced by differentially expressed genes are consistent with MKD characteristics. Top-scoring diseases based on the *p*-value are indeed dermatological diseases and conditions (*p*-value 9.40 × 10^−3^– 1.98 × 10^−14^, 35 genes assigned), immunological diseases (*p*-value 1.29 × 10^−2^– 1.98 × 10^−14^, 82 genes), organismal injury and abnormalities (*p*-value 1.41 × 10^−2^– 1.98 × 10^−14^, 181 genes), infectious diseases (*p*-value 9.40 × 10^−3^– 9.83 × 10^−8^, 71 genes), and inflammatory diseases (*p*-value 1.22 × 10^−2^ –9.83 × 10^−8^, 73 genes) (Figure 2A). Predicted decreased functions with both significant *p*-value and z-score are viral infection (*p*-value 1.05 × 10^−6^, 70 genes), quantity of blood cells (*p*-value 1.62 × 10^−4^, 43 genes), quantity of hematopoietic progenitor cells (*p*-value 2.22 × 10^−4^, 23 genes), cell death of connective tissue cells (*p*-value 2.34 × 10^−4^, 28 genes), quantity of lymphatic system cells (*p*-value, 1.89 × 10-3, 31 genes), and quantity of reticulocytes (*p*-value 2.58 × 10^−3^, 5 genes) (Figure 2B).

RNA post-transcriptional modification (*p*-value 1.52 × 10^−3^–5.18 × 10^−7^, 22 genes), cell death and survival (*p*-value 1.41 × 10^−2^–2.94 × 10-^6^, 96 genes), cell-to-cell signaling and interaction (*p*-value 1.40 × 10^−2^–3.04 × 10, 50 genes), cellular development (*p*-value 1.33 × 10^−2^–3.79 × 10^−5^, 87 genes), and cellular compromise (*p*-value 1.29 × 10^−2^–5.56 × 10-^5^, 32 genes) are the top-5 significantly enriched key molecular and cellular functions influenced by differentially expressed genes. High scoring functions and development related to physiological systems include cardiovascular system development and function (*p*-value 1.33 × 10^−2^–3.04 × 10^−6^, 58 genes), hematopoiesis (*p*-value 1.33 × 10^−2^–3.79 × 10^−5^, 44 genes), tissue development (*p*-value 1.36 × 10^−2^–3.79 × 10^−5^, 68 genes), hematological system development and function (*p*-value 1.38 × 10^−2^–6.06 × 10^−5^, 76 genes), and immune cell trafficking (*p*-value 1.38 × 10^−2^–6.06 × 10^−5^, 43 genes).

### 3.4. Canonical Pathways and Interaction Network Analysis of Differentially Expressed Genes

**Figure 3:**
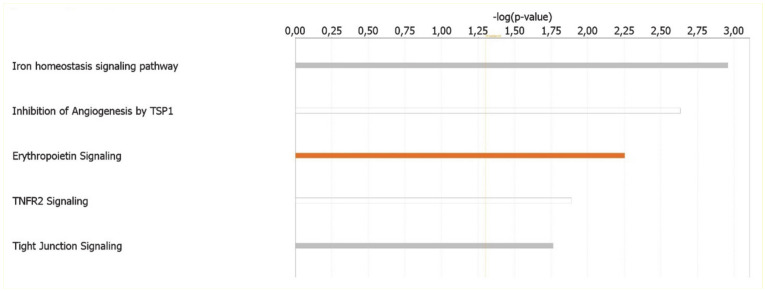
Ingenuity Pathway Analysis: Illustrated are the top canonical pathways most significantly associated with differentially expressed genes in MKD patients. The color of the bars refers to the prediction of activation (orange bars) or inhibition (blue bars) based on the z score. The bars are grey if the prediction cannot be assessed and white when z = 0.

The canonical pathways analysis led to the recognition of the key signaling pathways in which the differentially expressed genes could be involved. A total of 24 pathways significantly associated to MKD (*p* < 0.05) have been identified. Significance values have been calculated based on the Fisher’s right tailed exact test. The iron homeostasis signaling pathway (*p*-value 1.10 × 10^−3^), the inhibition of angiogenesis by thrombospondin 1 (TSP1) (*p*-value 2.32 × 10^−3^), erythropoietin signaling (*p*-value 5.59 × 10^−3^), Tumor necrosis factor receptor 2 (TNFR2) signaling (*p*-value 1.29 × 10^−2^), and tight junction signaling (*p*-value 1.72 × 10^−2^) are the five major pathways identified by IPA analysis (Figure 3).

The molecular interaction network of the identified genes differentially expressed in MKD was subsequently generated by IPA. Fifteen networks were identified and were ranked by the score, from 10 to 63. The most enriched networks were RNA Post-Transcriptional Modification, RNA Damage and Repair, Molecular Transport (score = 63), Hematological System Development and Function, Hematopoiesis (score = 44), Neurological Disease, Cell Death and Survival, Cellular Compromise (score = 34), Cell Morphology, Cellular Assembly and Organization, Cellular Development (score = 27), Protein Synthesis, Protein Degradation, Cellular Compromise (score = 27).

### 3.5. Anemia and Inflammation Networks

**Figure 4:**
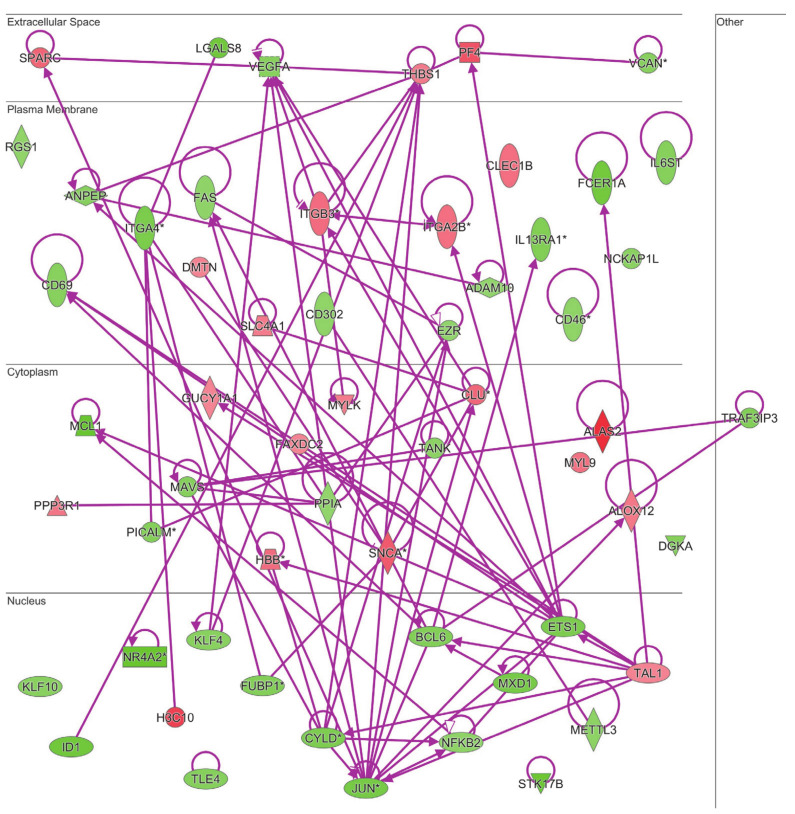
Connectivity of differentially expressed genes in the context of an anemia network. Upregulated genes are depicted by geometric symbols in red, downregulated ones are green. The color intensity is directly related to the fold change value. Continuous lines refer to the genes that are directly interconnected. The * indicates genes identified by more than one probe in the microarray.

Both the gene ontology and ingenuity pathway analyses above reported highlighted hematological system and hematopoiesis as linked biological functions and pathways in which differentially expressed genes in MKD could be involved. Since anemia is a common clinical feature in MKD patients, never analyzed at the molecular level in the context of MKD, we sought to examine the direct and indirect relationships among our dataset of genes in the context of anemia by IPA. The resulting network identifies 60 differentially expressed genes associated to anemia in MKD patients, among which 40 downregulated and 20 upregulated (Figure 4).

**Figure 5:**
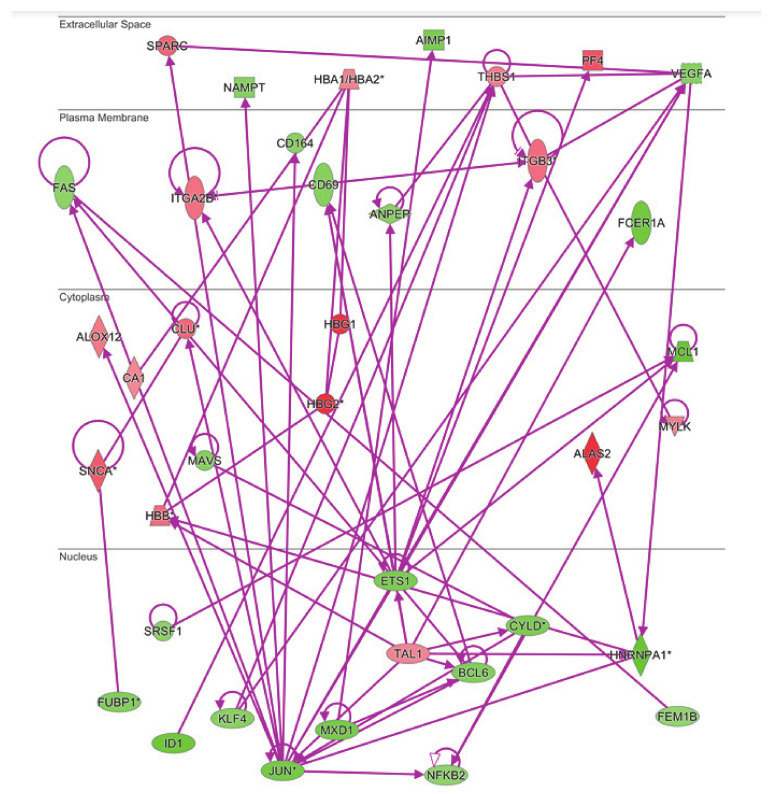
Connectivity of differentially expressed genes in the context of inflammation network. The * indicates genes identified by more than one probe in the microarray.

**Figure 6:**
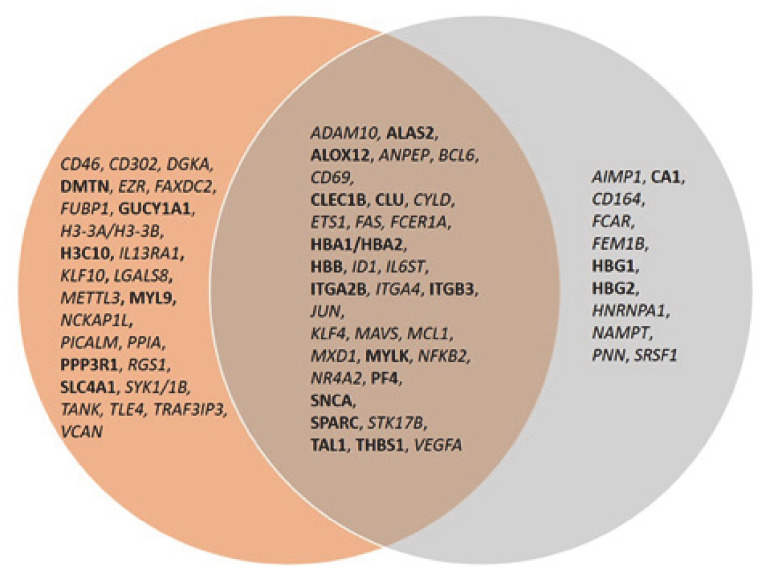
Venn diagram of common genes among the anemia and inflammation networks. Upregulated genes are in bold font, downregulated genes in italic.

The same analysis was done to examine the connectivity of up and downregulated genes from the MKD dataset in the context of the inflammatory process, that is at the basis of the autoinflammatory condition. The network in Figure 5 shows the direct connections among 45 MKD differentially expressed genes, 28 downregulated and 17 upregulated. Noteworthy, 34 of the genes differentially expressed in MKD are in common among the anemia and the inflammation networks, suggesting an overlap among these networks with potential functional implications in MKD (Figure 6).

### 3.6. Upstream Regulator Analysis

We then analyzed upstream regulators to find which molecules and transcription factors are more likely responsible for the changes observed in gene expression in PBMC from MKD patients. IPA upstream regulator analysis led to the identification of several upstream regulators with both significant *p*-value and z-score for predicted activation/inhibition. The overlap *p*-value indicates whether the overlapping among differentially expressed genes in the dataset and targets of the transcriptional regulator identified is significant (Table 3). Among the top 5 upstream regulators GATA1, erythropoietin (EPO), Bromodomain and Extra-Terminal motif (BET), and Runt-related transcription factor 1 (RUNX1) were predicted to be activated whereas Signal Transducer And Activator Of Transcription 3 (STAT3) was predicted to be inhibited. All these upstream regulators are known to be involved in physiological and dysregulated hematopoiesis.

## 4. Discussion

Omics approaches can be extremely informative, promoting the identification of molecular mechanisms underlying a pathology and hence the discovery of novel biomarkers for diagnosis, prognosis, and treatment. In our study, we analyzed a dataset deposited in a public repository, regarding the gene expression profiling of PBMC from patients with several autoinflammatory periodic syndromes, including a subgroup of MKD patients [15,18]. Considering the rarity of MKD disease and the presence of matched controls, this dataset is particularly precious and informative, and can be very useful to lead to novel information and make new hypotheses about the molecular mechanisms accounting for this rare disease. Our study is the first to report an overall bioinformatic analysis focused on MKD patients versus children matched controls and led to the identification of the most significant differentially expressed genes and their predicted relationships into biological processes, pathways, and networks highly relevant for MKD physiopathology.

Our bioinformatic analysis of MKD patients revealed the hematopoiesis, and in particular erythrocytes development, as a predominant biological function deregulated in affected patients with respect to control subjects. This is in line with the clinical observations of anemia made in case reports of MKD. Indeed, hematological abnormalities are always present in MKD patients along with autoinflammatory manifestations, so much so that they are often treated as hematological patients before the conclusive diagnosis of MKD. To date, there are no available studies about the molecular mechanisms underlaying hematological alterations in MKD. Anemia is diagnosed when the number of red blood cells or the levels of hemoglobin (Hb) are inadequate to sustain the physiologic demand. Physiologically, erythropoiesis provides novel erythrocytes, through a finely balanced and regulated process between medullary production and elimination of senescent ones by spleen and liver. Several acute and chronic stresses, including infection and inflammation, have been reported to affect erythrocyte development and function through the block of medullary steady-state erythropoiesis, leading to anemia. In these cases, an alternative erythropoiesis process, named stress erythropoiesis, is activated to preserve homeostasis until the removal of stress and the restoration of steady-state erythropoiesis [19]. The progenitors and the signals that drive stress erythropoiesis are different from steady-state erythropoiesis, as well as its putative site. Indeed, stress erythropoiesis occurs in extramedullary sites (spleen and liver) from immature stress erythropoietic progenitors migrated from the bone marrow. It has been reported that induction of stress erythropoiesis in anemic mice is associated with splenomegaly, augmented iron uptake and increase in the number of erythroblasts [20]. In case of inflammation, the pro-inflammatory signals at the same time inhibit medullary erythropoiesis in favor of myelopoiesis to sustain the proper inflammatory response and stimulate waves of extramedullary stress erythropoiesis to preserve red blood cells homeostasis. The balance is extremely fragile, also considering that inflammation shortens erythrocytes survival increasing their turnover, so that in chronic inflammatory conditions anemia consequently occurs. It has been estimated that inflammation contributes up to 40% cases of anemia, known as anemia of inflammation or of chronic disease [21]. Recurrent and spontaneous inflammatory flares affecting various parts of the body, characterize MKD in a similar way to other systemic chronic autoinflammatory disorders, with hyperproduction of proinflammatory cytokines among whose IL1β is the most critical one. The hematological manifestations observed in MKD patients, namely anemia, lymphadenopathy, and splenomegaly, are common features also in other autoinflammatory disorders, arguing in favor of their close dependence and correlation to the inflammatory process. In several MKD case reports anemia is generally described as moderate to severe normocytic anemia with low Hb levels, high number of circulating nucleated red blood cells, and fewer reticulocytes. Bone marrow aspirates performed in some patients displayed dyserythropoiesis of both erythroid and myeloid lineage, hypercellularity, block of medullary erythropoiesis at the normoblast stage, and dysplasia. Erythropoietin levels, when measured, were elevated, whereas only some patients also showed iron deficiency and elevated ferritin. Anemia is observed in association to hepatosplenomegaly, particularly during the febrile attacks, and improves with the pharmacological therapy in responding patients [8,12,22,23,24,25,26]. These features are suggestive of inadequate medullary steady state erythropoiesis and provide direct (through liver biopsies) and indirect proof of extramedullary stress erythropoiesis in MKD. Our functional and network analyses of statistically significant gene expression changes predicted the activation of EPO signaling as the third most significant key pathway in which differentially expressed genes in MKD could be involved. It is known that hypoxia occurring during anemia elicits production and release of EPO from the kidney and consequent extramedullary erythropoiesis in the spleen to try to compensate the anemic condition [27]. EPO signaling regulates a different set of genes in stress erythropoiesis with respect to steady-state erythropoiesis, prompting erythroid differentiation [28]. Under chronic stress, EPO inhibits the proapoptotic gene *FAS*, which we found downregulated in MKD patients, to prevent erythroblast apoptosis, as observed also in β-thalassemia [29]. Moreover, EPO induces 5′-aminolevulinate synthase 2 (ALAS2), the key rate-limiting enzyme of the heme biosynthesis in erythroid cells, fundamental for correct hemoglobin formation. Through our analysis in the MKD dataset we found that ALAS2 is the most upregulated gene expressed in MKD patients, followed by Hemoglobin subunit gamma-1 (HBG1) and HBG2 transcripts encoding for gamma globin. The transcription factors involved in the expression of erythroid specific genes, including globin genes, are GATA1, the master regulator of both normal and aberrant erythropoiesis and its interactors like T-Cell Acute Lymphocytic Leukemia Protein 1 (TAL1), that is indeed overexpressed in the MKD dataset [30].

Fetal hemoglobin (HbF, α_2_γ_2_) transcripts were highly upregulated in MKD with respect to control children, like in genetic forms of anemia as the hemoglobinopathies thalassemia and fetal hemoglobin quantitative trait locus 1, that indeed are highly correlated with MKD on the basis of our gene ontology analysis. Under physiological conditions, HbF represents only a small fraction of total hemoglobin (1–5%) in adult erythrocytes, since gamma globin gene expression is usually repressed [31]. In sickle cell disease and β thalassemia, the derepression of gamma globin gene can compensate for the lack of adult hemoglobin (HbA, α_2_β_2_) through the production of HbF. It is important to note that stress erythropoiesis displays several similarities with fetal erythropoiesis. Indeed, both stress and fetal erythroid progenitors express high levels of gamma globin and hence of fetal hemoglobin. In stress erythropoiesis, rapid erythroid regeneration induces the release in the blood of early erythroid progenitors, which retain the molecular expression program of gamma globin gene, eliciting an increment in circulating HbF levels. Thus, it is reasonable that, in MKD, stress erythropoiesis releases into circulation not terminally differentiated and still nucleated cells that are able to express fetal globin, as part of the stress program. Since HbF has a higher oxygen affinity than HbA, it cannot compensate or even it contributes to the anemic condition. The high levels of carbonic anhydrase transcripts (CA1) in our dataset led us to exclude the possibility of a reversion to a fetal form of erythropoiesis in MKD, similarly to thalassemia patients. However, the specific molecular mechanisms at the basis of such stress induced Hb switch in MKD patients remain to be determined.

MKD patients have fewer reticulocytes in circulation. Fundamental for the release of reticulocytes into the bloodstream and for their subsequent differentiation into functional mature biconcave erythrocytes are the enucleation process and the following membrane/cytoskeleton rearrangement. The process of enucleation is like an asymmetric cytokinesis and involves similar mechanisms and molecules, as microtubules polarization, contractile actomyosin ring formation, and vesicle trafficking. Rac GTPases, which act by regulating both actin polymerization and lipid rafts organization, are fundamental for enucleation [32]. It is well known that Rac, belonging to the Rho family of small GTPases, along with Rab and Ras families, requires prenylation for proper attachment to the membrane, localization to specific cell compartments, and interaction with partners and effectors. In MKD, as a consequence of the enzymatic deficiency of MVK in the mevalonate pathway, the low availability or lack of the isoprenoid moieties (farnseylpyrophosphate and geranylgeranylpyrophosphate) compromises the correct localization and functions of prenylated proteins, thus affecting, among the other biological functions, erythropoiesis and causing anemia. Several proteins of the Rho family (RhoA, Rac, Cdc42) have been reported to play fundamental roles in erythroid maturation and the enucleation process [33]. Inhibition or lack of the Rho effector mDia2, which drives actin nucleation and is highly expressed in erythroblasts, has been reported to lead to their defective enucleation [34]. Pharmacological inhibition of Rho-associated kinase 1 (ROCK1) also inhibited enucleation [35]. It is interesting to note that a form of congenital dyserythropoietic anemia type III is due to a mutation in the Kinesin-like protein (KIF23) gene, encoding for kinesin family member 23, that is essential for Rho-mediated cytokinesis [36]. Mice lacking Rac1 or Rac2 have been reported to suffer from hemolytic anemia caused by alterations in the erythrocyte actin cytoskeleton with greater mechanical fragility of the erythrocyte and reduced ability to adapt shape [37]. The observed increase, in the MKD dataset, in the expression levels of transcripts encoding for proteins that form the erythrocyte spectrin-based membrane skeleton connected to actin, like Solute Carrier Family 4 Member 1 (SLC4A1) and the actin-binding protein dematin, could be a consequence of defective prenylation of Rho and Rac proteins and could perturb the structural integrity of the membrane and hence erythrocyte cell deformability, contributing to anemia. All these aspects deserve further investigation at the cellular and molecular level in the context of MKD to better understand the molecular mechanisms underlaying the hematological alterations observed in this pathology.

Terminal differentiation of erythrocytes takes place in erythroblastic islands. In particular, stress erythropoiesis is highly dependent on the interactions of stress erythroid progenitors with macrophages into the erythroblastic islands and with fibronectin in the microenvironment. Several adhesion molecules are implied in their reciprocal interaction. Integrins are instrumental for both erythroid proliferation and differentiation. In particular, α4 integrins, which are expressed on erythroid cells, have been reported to affect terminal erythroid maturation under stress. A significant impairment of stress response is observed in the absence of α4 integrin [38,39]. This is consistent with our observation of lower levels of integrin α4 in PBMC from MKD patients. On the other hand, αIIbβ3 integrins that were upregulated in MKD patients can interact with ICAM-4 on red blood cells and have been reported to be involved in their production and release from the erythropoietic island [40].

Numerous chronic inflammatory and autoinflammatory diseases, characterized by hypersecretion of IL1 cytokines, present hematological alterations, including anemia. Indeed, inhibition of lymphopoiesis and erythropoiesis with concomitant stimulation of myeloid cells production by IL1β, has been observed in several models [41,42]. Chronic exposure to IL1β harms both self-renewal and function of hematopoietic stem cells in a reversible manner. This implies that IL1β signaling blockade through anakinra, canakinumab and other biologicals used for the treatment of a broad number of inflammatory diseases, including MKD has, as a potential secondary benefit, the recovery of haematological function, that has indeed been clinically observed [43].

Chronic IL1β exposure has been also reported to stimulate megakaryopoiesis [44]. This is consistent with our observations in MKD patients where a number of upregulated genes downstream RUNX1 transcription factor (e.g., Arachidonate 12-Lipoxygenase (ALOX12), Myosin regulatory light polypeptide 9, MYL9) are involved in megakaryocyte differentiation, or in platelet activation and aggregation, blood coagulation, and hemostasis, all processes associated to the inflammatory response.

Obviously, we are aware that our analysis suffers from certain limitations due to the design of the microarray experiment that, being performed on PBMC, excluded mature anucleate erythrocytes. Indeed, even though until recently it was thought that anucleate erythrocytes were RNA-free, now we know that their transcriptome regulates their development and function under physiological and pathological conditions [45]. Noteworthy, the changes in single cell populations of peripheral blood that vary in the disease context like the increment in immature nucleated erythrocytes, are informative about the hematological abnormalities observed and are perfectly reflected at the molecular level by the differences reported in gene expression among patients and controls. However, we can only infer and not ascribe for sure the variation of the expression of a specific gene to a certain cell population of the peripheral blood, unless it is cell-type specific. It is also important to note that the patients included in the dataset are in different stages of the disease (active, inactive, in flare) and subjected to different therapeutic regimens and their relative small number, given the rarity of the disease, did not allow a stratification in subgroups for the analysis [15].

## 5. Conclusions

Coming to a diagnosis of MKD can be extremely difficult before genetic testing is done, since IgD levels in plasma and mevalonic acid levels in urine are not always elevated. Moreover, also considering the broad spectrum of clinical manifestations, the clinical phenotype can be challenging to be identified and especially the presence of hematological alterations can be a confounding element toward the diagnosis of a myelodysplastic syndrome, or a hematological disorder derived by congenital infection. For these reasons misdiagnoses and delayed diagnosis are frequent for MKD patients. Understanding molecular bases of MKD can thus help to facilitate its diagnosis by clinicians and to improve its management and pharmacological treatment.

In our study, we found that the autoinflammatory process in MKD patients, similarly to what has been observed in other pathologies characterized by chronic inflammation, may contribute to dysregulated hematopoietic homeostasis and consequent hematological abnormalities.

Further studies on the relevance of hematopoietic changes in the context of MKD and on their mechanisms are thus worth of investigation.

## Figures and Tables

**Table 1:** Top 25 upregulated genes in mevalonate kinase deficiency (MKD) patients (*p*-value < 0.05, log_2_ FC ≥ 1.4).

Symbol	Entrez Gene Name	Average Log_2_ FC	Adj. *p*-Value
ALAS2	5′-aminolevulinate synthase 2	3.560	4.28 × 10^−6^
HBG2	hemoglobin subunit gamma 2	3.035	2.60 × 10^−5^
HBG1	hemoglobin subunit gamma 1	2.930	2.52 × 10^−5^
IFI27	interferon alpha inducible protein 27	2.910	7.36 × 10^−4^
GNG11	G protein subunit gamma 11	2.740	3.00 × 10^−8^
HBD	hemoglobin subunit delta	2.560	4.90 × 10^−4^
PF4V1	platelet factor 4 variant 1	2.500	3.86 × 10^−6^
H3C10	H3 clustered histone 10	2.490	1.91 × 10^−9^
GMPR	guanosine monophosphate reductase	2.420	3.52 × 10^−8^
GP1BB	glycoprotein Ib platelet subunit beta	2.340	5.37 × 10^−7^
AHSP	alpha hemoglobin stabilizing protein	2.330	7.27 × 10^−5^
PF4	platelet factor 4	2.170	8.59 × 10^−9^
H2AC8	H2A clustered histone 8	2.140	3.23 × 10^−9^
SCD5	stearoyl-CoA desaturase 5	2.120	9.67 × 10^−4^
HBQ1	hemoglobin subunit theta 1	2.050	8.14 × 10^−7^
SNCA	synuclein alpha	2.045	2.97 × 10^−6^
MLH3	mutL homolog 3	2.023	1.61 × 10^−7^
PDZK1IP1	PDZK1 interacting protein 1	1.930	9.35 × 10^−6^
SOX15	SRY-box transcription factor 15	1.920	9.48 × 10^−12^
SPARC	secreted protein acidic and cysteine rich	1.890	1.20 × 10^−5^
CLU	clusterin	1.867	2.52 × 10^−6^
TRIM58	tripartite motif containing 58	1.850	1.15 × 10^−5^
SELENBP1	selenium binding protein 1	1.820	2.88 × 10^−4^
SYNE1	spectrin repeat containing nuclear envelope protein 1	1.810	4.70 × 10^−8^
ITGB3	integrin subunit beta 3	1.795	1.42 × 10^−3^

**Table 2:** Top 25 downregulated genes in MKD patients (*p*-value < 0.05, log_2_ FC ≤ 1.4).

Symbol	Entrez Gene Name	Average Log_2_ FC	Adj. *p*-Value
EIF1	eukaryotic translation initiation factor 1	−3.620	2.01 × 10^−14^
NEAT1	nuclear paraspeckle assembly transcript 1	−2.610	3.57 × 10^−12^
RAD23B	RAD23 homolog B, nucleotide excision repair protein	−2.520	7.97 × 10^−10^
MCL1	MCL1 apoptosis regulator, BCL2 family member	−2.440	3.64 × 10^−6^
SLC16A3	solute carrier family 16 member 3	−2.420	2.46 × 10^−10^
NR4A2	nuclear receptor subfamily 4 group A member 2	−2.400	8.52 × 10^−6^
HNRNPA1	heterogeneous nuclear ribonucleoprotein A1	−2.390	1.01 × 10^−6^
STK17B	serine/threonine kinase 17b	−2.250	1.11 × 10^−8^
C6orf62	chromosome 6 open reading frame 62	−2.200	2.27 × 10^−8^
ID1	inhibitor of DNA binding 1, HLH protein	−2.120	2.21 × 10^−6^
LGALS8	galectin 8	−2.110	8.57 × 10^−9^
SPTSSA	serine palmitoyltransferase small subunit A	−2.110	2.24 × 10^−7^
FCER1A	Fc fragment of IgE receptor Ia	−2.070	1.20 × 10^−5^
CLEC2D	C-type lectin domain family 2 member D	−2.070	8.15 × 10^−8^
HNRNPD	heterogeneous nuclear ribonucleoprotein D	−2.030	5.27 × 10^−6^
KLF9	Kruppel like factor 9	−2.030	6.84 × 10^−8^
G0S2	G0/G1 switch 2	−2.020	1.64 × 10^−3^
HSPB11	heat shock protein family B (small) member 11	−2.020	3.80 × 10^−8^
JUN	Jun proto-oncogene, AP-1 transcription factor subunit	−1.985	7.48 × 10^−6^
GOLGA8A/8B	golgin A8 family member A	−1.967	8.77 × 10^−6^
POLI	DNA polymerase iota	−1.960	1.62 × 10^−6^
CDV3	CDV3 homolog	−1.950	3.47 × 10^−7^
MXD1	MAX dimerization protein 1	−1.950	5.88 × 10^−9^
HNRNPH1	heterogeneous nuclear ribonucleoprotein H1	−1.940	2.05 × 10^−6^
AHSA2P	activator of HSP90 ATPase homolog 2, pseudogene	−1.920	6.44 × 10^−6^
MED6	mediator complex subunit 6	−1.900	2.72 × 10^−6^

**Table 3:** Top five upstream regulators.

Upstream Regulator	Molecule Type	Predicted Activation State	*p*-Value of Overlap	Target Molecules in Dataset
GATA1	transcription regulator	Activated	1.11 × 10^−10^	AHSP, ALAS2, ALOX12, CA1, CD164, CPD, DMTN, ETS1, FCER1A, GP1BB, HBA1/HBA2, HBB, HBD, HBG1, HBG2, IL6ST, ITGA2B, ITGB3, NR4A2, PF4, SLC4A1, SNCA, TAL1
EPO	cytokine	Activated	1.34 × 10^−6^	ALAS2, BCL6, CA1, FAS, HBA1/HBA2, HBB, HBG2, ID1, ITGA4, JUN, MCL1, NACA, SDHC, SLC4A1, TAL1, TSN, VEGFA
BET	group	Activated	4.25 × 10^−5^	ALAS2, DMTN, SLC4A1, TAL1
RUNX1	transcription regulator	Activated	6.65 × 10^−5^	ALAS2, ALOX12, FAS, HBA1/HBA2, HBG2, ITGA2B, ITGA4, ITGB3, MYL9, PF4, SLC4A1, TAL1, VEGFA
STAT3	transcription regulator	Inhibited	9.19 × 10^−5^	AHSP, ALAS2, ALDH1A1, BCL6, CD46, CTTN, FAS, HBB, HBG1, HNRNPD, ID1, IFI27, IL6ST, KLF4, MCL1, NAMPT, NFKB2, NR4A2, THBS1, UPF3A, VCAN, VEGFA

## Data Availability

The data presented in this study are openly available in GEO (https://www.ncbi.nlm.nih.gov/geo/), reference number [GSE43553. Balow, J.E., Ryan, J.G., Chae, J.J., Booty, M.G., Bulua, A., Stone, D., Sun, H., Greene, J., Barham, B., Goldbach-Mansky, R., Kastner, D.L., Aksentijevich, I. 2013. Microarray-based gene expression profiling in patients with cryopyrin-associated periodic syndromes defines a disease-related signature and IL-1-responsive transcripts].

## Supplementary Materials

The following are available online at https://www.mdpi.com/1660-4601/18/3/1170/s1, Supplementary Table S1.

## Abbreviations

Mevalonate kinase deficiency (MKD); type D hyperimmunoglobulinemia (HIDS); mevalonic aciduria (MA); Erythrocyte sedimentation rate (ESR); C-reactive protein (CRP); creatine kinase (CK); interleukin 1 beta (IL1β); recombinant human IL1 receptor a (rhIL1Ra); tumor necrosis factor (TNF); Non-steroidal anti-inflammatory drugs (NSAIDs); peripheral blood mononuclear cells (PBMC); false discovery rate (FDR); Ingenuity Pathway Analysis (IPA); thrombospondin 1 (TSP1); Tumor necrosis factor receptor 2 (TNFR2); erythropoietin (EPO); Bromodomain and Extra-Terminal motif (BET); Runt-related transcription factor 1 (RUNX1); Signal Transducer And Activator Of Transcription 3 (STAT3); hemoglobin (Hb); 5′-aminolevulinate synthase 2 (ALAS2); Hemoglobin subunit gamma-1 (HBG1); T-Cell Acute Lymphocytic Leukemia Protein 1 (TAL1); Fetal hemoglobin (HbF); Rho-associated kinase 1 (ROCK1); Solute Carrier Family 4 Member 1 (SLC4A1); Arachidonate 12-Lipoxygenase (ALOX12); Myosin regulatory light polypeptide 9, MYL9.
