# Supplementary material for: Gene Expression Analysis of Mevalonate Kinase Deficiency Affected Children Identifies Molecular Signatures Related to Hematopoiesis
Source: Int J Environ Res Public Health. 2021 Jan 28;18(3):1170. doi: 10.3390/ijerph18031170 (PMC7908123; doi:10.3390/ijerph18031170)
Supplement: Supplementary file 1 [file ijerph-18-01170-s001.pdf]

Supplementary table S1. List of differentially expressed transcripts (FDR adjusted *p*-value < 0.05 and −1.4 ≤ FC ≥1.4).

ID	Symbol	Entrez Gene Name	Adj. *p*-Value	Log2 FC
214895_s_at	ADAM10	ADAM metallopeptidase domain 10	3,11E-05	−1,400
205997_at	ADAM28	ADAM metallopeptidase domain 28	6,57E-05	−1,400
220606_s_at	ADPRM	ADP-ribose/CDP-alcohol diphosphatase, manganese dependent	6,50E-06	−1,430
217410_at	AGRN	agrin	2,34E-10	1,420
212980_at	AHSA2P	activator of HSP90 ATPase homolog 2, pseudogene	6,44E-06	−1,920
219672_at	AHSP	alpha hemoglobin stabilizing protein	7,27E-05	2,330
202541_at	AIMP1	aminoacyl tRNA synthetase complex interacting multifunctional protein 1	4,91E-06	−1,830
210269_s_at	AKAP17A	A-kinase anchoring protein 17A	2,64E-10	−1,560
211560_s_at	ALAS2	5'-aminolevulinate synthase 2	4,28E-06	3,560
212224_at	ALDH1A1	aldehyde dehydrogenase 1 family member A1	8,93E-04	−1,400
205583_s_at	ALG13	ALG13 UDP-N-acetylglucosaminyltransferase subunit	9,50E-07	−1,430
207206_s_at	ALOX12	arachidonate 12-lipoxygenase, 12S type	4,76E-05	1,630
208498_s_at	AMY1C (includes others)	amylase alpha 1C	3,83E-05	−1,700
201043_s_at	ANP32A	acidic nuclear phosphoprotein 32 family member A	5,61E-09	−1,760
202888_s_at	ANPEP	alanyl aminopeptidase, membrane	7,40E-04	−1,600
221013_s_at	APOL2	apolipoprotein L2	6,57E-11	1,600
219094_at	ARMC8	armadillo repeat containing 8	3,47E-08	−1,710
207798_s_at	ATXN2L	ataxin 2 like	2,16E-07	−1,410
215990_s_at	BCL6	BCL6 transcription repressor	1,74E-07	−1,700
200776_s_at	BZW1	basic leucine zipper and W2 domains 1	1,09E-06	−1,570
222309_at	C6orf62	chromosome 6 open reading frame 62	2,27E-08	−2,200
220038_at	C8orf44-SGK3/SGK3	serum/glucocorticoid regulated kinase family member 3	1,17E-03	−1,450
205950_s_at	CA1	carbonic anhydrase 1	2,37E-03	1,470
217814_at	CCDC47	coiled-coil domain containing 47	1,02E-09	−1,490
204645_at	CCNT2	cyclin T2	6,17E-07	−1,700
208653_s_at	CD164	CD164 molecule	3,30E-06	−1,560
203799_at	CD302	CD302 molecule	5,18E-05	−1,470
211574_s_at	CD46	CD46 molecule	4,75E-06	−1,550
207549_x_at	CD46	CD46 molecule	1,68E-05	−1,420
209795_at	CD69	CD69 molecule	1,03E-05	−1,610
203377_s_at	CDC40	cell division cycle 40	5,62E-07	−1,490
213548_s_at	CDV3	CDV3 homolog	3,47E-07	−1,950
203493_s_at	CEP57	centrosomal protein 57	2,86E-05	−1,490
204482_at	CLDN5	claudin 5	2,85E-07	1,480
220496_at	CLEC1B	C-type lectin domain family 1 member B	1,06E-05	1,770
220132_s_at	CLEC2D	C-type lectin domain family 2 member D	8,15E-08	−2,070
214252_s_at	CLN5	CLN5 intracellular trafficking protein	3,99E-09	−1,520
222043_at	CLU	clusterin	1,40E-07	1,640
208791_at	CLU	clusterin	2,66E-06	1,950
208792_s_at	CLU	clusterin	4,77E-06	2,010
214336_s_at	COPA	COPI coat complex subunit alpha	2,29E-06	−1,460
213758_at	COX4I1	cytochrome c oxidase subunit 4I1	1,47E-06	−1,440
201942_s_at	CPD	carboxypeptidase D	3,15E-08	−1,500
202979_s_at	CREBZF	CREB/ATF bZIP transcription factor	2,37E-06	−1,680
221139_s_at	CSAD	cysteine sulfinic acid decarboxylase	2,53E-09	−1,460

ID	Symbol	Entrez Gene Name	Adj. *p*-Value	Log2 FC
201906_s_at	CTDSPL	CTD small phosphatase like	1,40E-05	1,470
201059_at	CTTN	cortactin	2,86E-05	1,550
214073_at	CTTN	cortactin	1,30E-06	1,910
215997_s_at	CUL4B	cullin 4B	2,32E-12	−1,780
222142_at	CYLD	CYLD lysine 63 deubiquitinase	7,20E-08	−1,850
60084_at	CYLD	CYLD lysine 63 deubiquitinase	2,37E-07	−1,760
214630_at	CYP11B2	cytochrome P450 family 11 subfamily B member 2	1,50E-09	1,550
212514_x_at	DDX3X	DEAD-box helicase 3 X-linked	7,04E-07	−1,520
211272_s_at	DGKA	diacylglycerol kinase alpha	1,85E-07	−1,640
212105_s_at	DHX9	DExH-box helicase 9	2,98E-07	−1,600
215210_s_at	DLST	dihydrolipoamide S-succinyltransferase	3,55E-11	−1,400
204505_s_at	DMTN	dematin actin binding protein	3,69E-06	1,500
221782_at	DNAJC10	DnaJ heat shock protein family (Hsp40) member C10	1,09E-07	−1,740
221781_s_at	DNAJC10	DnaJ heat shock protein family (Hsp40) member C10	5,34E-08	−1,610
215252_at	DNAJC7	DnaJ heat shock protein family (Hsp40) member C7	1,66E-06	−1,530
212225_at	EIF1	eukaryotic translation initiation factor 1	2,01E-14	−3,620
201123_s_at	EIF5A	eukaryotic translation initiation factor 5A	8,93E-04	−1,760
206338_at	ELAVL3	ELAV like RNA binding protein 3	4,52E-12	1,460
217294_s_at	ENO1	enolase 1	1,28E-03	−1,490
214447_at	ETS1	ETS proto-oncogene 1, transcription factor	8,65E-09	−1,840
218748_s_at	EXOC5	exocyst complex component 5	6,67E-05	−1,410
208621_s_at	EZR	ezrin	1,21E-04	−1,410
207475_at	FABP2	fatty acid binding protein 2	1,64E-10	1,460
214945_at	FAM153A/FAM153B	family with sequence similarity 153 member B	2,00E-06	−1,650
215719_x_at	FAS	Fas cell surface death receptor	4,22E-05	−1,460
220751_s_at	FAXDC2	fatty acid hydroxylase domain containing 2	1,20E-05	1,490
207674_at	FCAR	Fc fragment of IgA receptor	1,46E-05	-1,490
211734_s_at	FCER1A	Fc fragment of IgE receptor Ia	1,20E-05	−2,070
215567_at	FCF1	FCF1 rRNA-processing protein	3,00E-07	−1,810
212374_at	FEM1B	fem-1 homolog B	4,59E-08	−1,400
212847_at	FUBP1	far upstream element binding protein 1	2,22E-05	−1,830
214093_s_at	FUBP1	far upstream element binding protein 1	5,69E-05	−1,560
213524_s_at	G0S2	G0/G1 switch 2	1,64E-03	−2,020
204187_at	GMPR	guanosine monophosphate reductase	3,52E-08	2,420
204115_at	GNG11	G protein subunit gamma 11	3,00E-08	2,740
208798_x_at	GOLGA8A/GOLGA8B	golgin A8 family member A	2,61E-05	−1,700
210425_x_at	GOLGA8A/GOLGA8B	golgin A8 family member A	2,07E-07	−2,280
210424_s_at	GOLGA8A/GOLGA8B	golgin A8 family member A	9,92E-11	−1,920
206655_s_at	GP1BB	glycoprotein Ib platelet subunit beta	5,37E-07	2,340
221942_s_at	GUCY1A1	guanylate cyclase 1 soluble subunit alpha 1	1,92E-05	1,480
215071_s_at	H2AC6	H2A clustered histone 6	1,15E-06	1,550
214469_at	H2AC8	H2A clustered histone 8	3,23E-09	2,140
208579_x_at	H2BC12	H2B clustered histone 12	1,12E-06	1,460
202708_s_at	H2BC21	H2B clustered histone 21	8,62E-07	1,420
210387_at	H2BC8	H2B clustered histone 8	3,13E-06	1,540
211998_at	H3-3A/H3-3B	H3.3 histone A	6,62E-09	−1,690
206110_at	H3C10	H3 clustered histone 10	1,91E-09	2,490
210054_at	HAUS3	HAUS augmin like complex subunit 3	1,31E-06	−1,660
204018_x_at	HBA1/HBA2	hemoglobin subunit alpha 2	1,07E-02	1,500
217414_x_at	HBA1/HBA2	hemoglobin subunit alpha 2	9,76E-03	1,590

ID	Symbol	Entrez Gene Name	Adj. *p*-Value	Log2 FC
211696_x_at	HBB	hemoglobin subunit beta	5,44E-03	1,740
217232_x_at	HBB	hemoglobin subunit beta	6,36E-03	1,790
209116_x_at	HBB	hemoglobin subunit beta	6,22E-03	1,830
206834_at	HBD	hemoglobin subunit delta	4,90E-04	2,560
204848_x_at	HBG1	hemoglobin subunit gamma 1	2,52E-05	2,930
213515_x_at	HBG2	hemoglobin subunit gamma 2	4,50E-05	2,890
204419_x_at	HBG2	hemoglobin subunit gamma 2	6,94E-06	3,180
207361_at	HBP1	HMG-box transcription factor 1	9,76E-08	−1,460
220807_at	HBQ1	hemoglobin subunit theta 1	8,14E-07	2,050
205221_at	HGD	homogentisate 1,2-dioxygenase	3,60E-07	1,540
214308_s_at	HGD	homogentisate 1,2-dioxygenase	1,08E-09	1,650
210148_at	HIPK3	homeodomain interacting protein kinase 3	6,84E-08	−1,570
221919_at	HNRNPA1	heterogeneous nuclear ribonucleoprotein A1	2,51E-08	−2,460
222040_at	HNRNPA1	heterogeneous nuclear ribonucleoprotein A1	2,00E-06	−2,320
213359_at	HNRNPD	heterogeneous nuclear ribonucleoprotein D	5,27E-06	−2,030
212454_x_at	HNRNPDL	heterogeneous nuclear ribonucleoprotein D like	1,25E-04	−1,410
213470_s_at	HNRNPH1	heterogeneous nuclear ribonucleoprotein H1	2,05E-06	−1,940
214918_at	HNRNPM	heterogeneous nuclear ribonucleoprotein M	1,33E-04	−1,410
209512_at	HSDL2	hydroxysteroid dehydrogenase like 2	1,11E-07	−1,590
202558_s_at	HSPA13	heat shock protein family A (Hsp70) member 13	1,67E-06	−1,440
214163_at	HSPB11	heat shock protein family B (small) member 11	3,80E-08	−2,020
208937_s_at	ID1	inhibitor of DNA binding 1, HLH protein	2,21E-06	−2,120
202411_at	IFI27	interferon alpha inducible protein 27	7,36E-04	2,910
203819_s_at	IGF2BP3	insulin like growth factor 2 mRNA binding protein 3	3,62E-06	1,510
210904_s_at	IL13RA1	interleukin 13 receptor subunit alpha 1	1,89E-06	−1,890
211612_s_at	IL13RA1	interleukin 13 receptor subunit alpha 1	3,49E-06	−1,550
212196_at	IL6ST	interleukin 6 signal transducer	2,01E-07	−1,630
206494_s_at	ITGA2B	integrin subunit alpha 2b	5,74E-04	1,610
206493_at	ITGA2B	integrin subunit alpha 2b	1,12E-05	1,880
205885_s_at	ITGA4	integrin subunit alpha 4	1,27E-06	−1,860
205884_at	ITGA4	integrin subunit alpha 4	3,54E-06	−1,850
204627_s_at	ITGB3	integrin subunit beta 3	2,83E-03	1,560
204628_s_at	ITGB3	integrin subunit beta 3	1,61E-06	2,030
201464_x_at	JUN	Jun proto-oncogene, AP-1 transcription factor subunit	1,06E-06	−2,100
201466_s_at	JUN	Jun proto-oncogene, AP-1 transcription factor subunit	1,39E-05	−1,870
206765_at	KCNJ2	potassium inwardly rectifying channel subfamily J member 2	5,39E-05	1,440
202393_s_at	KLF10	Kruppel like factor 10	3,28E-06	−1,590
220266_s_at	KLF4	Kruppel like factor 4	2,46E-07	−1,580
203543_s_at	KLF9	Kruppel like factor 9	6,84E-08	−2,030
210690_at	KLRC4	killer cell lectin like receptor C4	5,66E-06	−1,700
210732_s_at	LGALS8	galectin 8	8,57E-09	−2,110
220121_at	LINS1	lines homolog 1	4,95E-08	−1,740
221211_s_at	MAP3K7CL	MAP3K7 C-terminal like	4,94E-06	1,570
203553_s_at	MAP4K5	mitogen-activated protein kinase kinase kinase kinase 5	1,13E-05	−1,400
215512_at	MARCHF6	membrane associated ring-CH-type finger 6	8,20E-08	−1,610
202654_x_at	MARCHF7	membrane associated ring-CH-type finger 7	1,51E-05	−1,730
220305_at	MAVS	mitochondrial antiviral signaling protein	2,33E-11	−1,630
200796_s_at	MCL1	MCL1 apoptosis regulator, BCL2 family member	3,64E-06	−2,440
207078_at	MED6	mediator complex subunit 6	2,72E-06	−1,900

ID	Symbol	Entrez Gene Name	Adj. *p*-Value	Log2 FC
213653_at	METTL3	methyltransferase like 3	9,66E-08	−1,500
214696_at	MIR22HG	MIR22 host gene	3,36E-07	−1,630
217216_x_at	MLH3	mutL homolog 3	4,05E-07	1,710
214525_x_at	MLH3	mutL homolog 3	4,38E-08	1,830
204838_s_at	MLH3	mutL homolog 3	3,31E-08	2,530
219822_at	MTRF1	mitochondrial translation release factor 1	4,57E-07	−1,420
206877_at	MXD1	MAX dimerization protein 1	5,88E-09	−1,950
201058_s_at	MYL9	myosin light chain 9	7,41E-04	1,740
202555_s_at	MYLK	myosin light chain kinase	3,12E-05	1,590
219378_at	NAA16	N-alpha-acetyltransferase 16, NatA auxiliary subunit	1,15E-04	−1,410
222018_at	NACA	nascent polypeptide associated complex subunit alpha	3,97E-08	−1,870
217739_s_at	NAMPT	nicotinamide phosphoribosyltransferase	4,55E-04	−1,590
216466_at	NAV3	neuron navigator 3	1,32E-05	1,400
209734_at	NCKAP1L	NCK associated protein 1 like	1,50E-12	−1,540
214657_s_at	NEAT1	nuclear paraspeckle assembly transcript 1	3,57E-12	−2,610
207535_s_at	NFKB2	nuclear factor kappa B subunit 2	1,80E-07	−1,470
215921_at	NPIPB4 (includes others)	nuclear pore complex interacting protein family member B4	1,95E-10	−1,440
216248_s_at	NR4A2	nuclear receptor subfamily 4 group A member 2	2,01E-06	−2,540
204621_s_at	NR4A2	nuclear receptor subfamily 4 group A member 2	7,64E-06	−2,470
204622_x_at	NR4A2	nuclear receptor subfamily 4 group A member 2	1,59E-05	−2,190
204081_at	NRGN	neurogranin	9,96E-07	1,630
212720_at	PAPOLA	poly(A) polymerase alpha	2,28E-06	−1,700
222035_s_at	PAPOLA	poly(A) polymerase alpha	1,86E-09	−1,630
222044_at	PCIF1	PDX1 C-terminal inhibiting factor 1	2,54E-09	−1,870
222045_s_at	PCIF1	PDX1 C-terminal inhibiting factor 1	3,39E-09	−1,560
205463_s_at	PDGFA	platelet derived growth factor subunit A	9,80E-06	1,570
219630_at	PDZK1IP1	PDZK1 interacting protein 1	9,35E-06	1,930
218319_at	PELI1	pellino E3 ubiquitin protein ligase 1	7,14E-07	−1,600
206390_x_at	PF4	platelet factor 4	8,59E-09	2,170
207815_at	PF4V1	platelet factor 4 variant 1	3,86E-06	2,500
215236_s_at	PICALM	phosphatidylinositol binding clathrin assembly protein	5,56E-06	−1,940
212511_at	PICALM	phosphatidylinositol binding clathrin assembly protein	1,11E-04	−1,410
219700_at	PLXDC1	plexin domain containing 1	4,28E-07	−1,610
212176_at	PNISR	PNN interacting serine and arginine rich protein	2,85E-05	−1,400
212036_s_at	PNN	pinin, desmosome associated protein	2,23E-06	−1,810
219317_at	POLI	DNA polymerase iota	1,62E-06	−1,960
214146_s_at	PPBP	pro-platelet basic protein	4,07E-06	1,610
217602_at	PPIA	peptidylprolyl isomerase A	4,43E-08	−1,410
204506_at	PPP3R1	protein phosphatase 3 regulatory subunit B, alpha	2,39E-12	1,670
217851_s_at	PRELID3B	PRELI domain containing 3B	3,65E-06	−1,460
221547_at	PRPF18	pre-mRNA processing factor 18	3,81E-06	−1,490
211090_s_at	PRPF4B	pre-mRNA processing factor 4B	2,45E-05	−1,720
202127_at	PRPF4B	pre-mRNA processing factor 4B	2,32E-05	−1,500
218683_at	PTBP2	polypyrimidine tract binding protein 2	6,08E-08	−1,520
200730_s_at	PTP4A1	protein tyrosine phosphatase 4A1	1,73E-05	−1,550
207791_s_at	RAB1A	RAB1A, member RAS oncogene family	1,20E-05	−1,660
209181_s_at	RABGGTB	Rab geranylgeranyltransferase subunit beta	1,97E-06	−1,690
213704_at	RABGGTB	Rab geranylgeranyltransferase subunit beta	3,11E-06	−1,580

ID	Symbol	Entrez Gene Name	Adj. *p*-Value	Log2 FC
207405_s_at	RAD17	RAD17 checkpoint clamp loader component	6,35E-07	−1,680
201222_s_at	RAD23B	RAD23 homolog B, nucleotide excision repair protein	7,97E-10	−2,520
212706_at	RASA4	RAS p21 protein activator 4	7,89E-10	−1,810
202988_s_at	RGS1	regulator of G protein signaling 1	1,85E-03	−1,430
213397_x_at	RNASE4	ribonuclease A family member 4	1,34E-04	−1,450
221430_s_at	RNF146	ring finger protein 146	5,07E-06	−1,410
221989_at	RPL10	ribosomal protein L10	2,34E-10	−1,670
214291_at	RPL17	ribosomal protein L17	1,80E-07	−1,550
213459_at	RPL37A	ribosomal protein L37a	5,09E-08	−1,400
216902_s_at	RRN3P1	RRN3 pseudogene 1	5,31E-08	−1,620
222310_at	SCAF4	SR-related CTD associated factor 4	5,13E-05	−1,450
220232_at	SCD5	stearoyl-CoA desaturase 5	9,67E-04	2,120
216591_s_at	SDHC	succinate dehydrogenase complex subunit C	3,33E-06	−1,480
201916_s_at	SEC63	SEC63 homolog, protein translocation regulator	1,28E-05	−1,500
214433_s_at	SELENBP1	selenium binding protein 1	2,88E-04	1,820
209769_s_at	SEPT5-GP1BB	SEPT5-GP1BB readthrough	9,86E-12	1,580
200986_at	SERPING1	serpin family G member 1	1,11E-02	1,650
221768_at	SFPQ	splicing factor proline and glutamine rich	4,03E-05	−1,690
208078_s_at	SIK1/SIK1B	salt inducible kinase 1	1,82E-05	−1,640
202856_s_at	SLC16A3	solute carrier family 16 member 3	1,02E-11	−2,760
202855_s_at	SLC16A3	solute carrier family 16 member 3	4,81E-10	−2,080
205592_at	SLC4A1	solute carrier family 4 member 1 (Diego blood group)	3,71E-04	1,610
203579_s_at	SLC7A6	solute carrier family 7 member 6	2,13E-07	−1,400
206875_s_at	SLK	STE20 like kinase	1,05E-07	−1,490
212927_at	SMC5	structural maintenance of chromosomes 5	3,85E-05	−1,400
210357_s_at	SMOX	spermine oxidase	8,01E-08	1,580
204467_s_at	SNCA	synuclein alpha	5,45E-06	1,990
204466_s_at	SNCA	synuclein alpha	4,99E-07	2,100
202113_s_at	SNX2	sorting nexin 2	2,96E-06	−1,680
217040_x_at	SOX15	SRY-box transcription factor 15	9,48E-12	1,920
200665_s_at	SPARC	secreted protein acidic and cysteine rich	1,20E-05	1,890
212460_at	SPTSSA	serine palmitoyltransferase small subunit A	2,24E-07	−2,110
201742_x_at	SRSF1	serine and arginine rich splicing factor 1	8,02E-05	−1,540
204299_at	SRSF10	serine and arginine rich splicing factor 10	3,89E-09	−1,740
210077_s_at	SRSF5	serine and arginine rich splicing factor 5	1,46E-09	−1,640
202817_s_at	SS18	SS18 subunit of BAF chromatin remodeling complex	2,00E-05	−1,600
214060_at	SSBP1	single stranded DNA binding protein 1	4,07E-08	−1,750
214597_at	SSTR2	somatostatin receptor 2	1,51E-10	1,490
205214_at	STK17B	serine/threonine kinase 17b	1,11E-08	−2,250
221638_s_at	STX16	syntaxin 16	8,83E-06	−1,710
215350_at	SYNE1	spectrin repeat containing nuclear envelope protein 1	4,70E-08	1,810
202840_at	TAF15	TATA-box binding protein associated factor 15	3,06E-11	−1,560
206283_s_at	TAL1	TAL bHLH transcription factor 1, erythroid differentiation factor	3,57E-05	1,490
210458_s_at	TANK	TRAF family member associated NFKB activator	2,62E-06	−1,520
207306_at	TCF15	transcription factor 15	2,97E-10	1,470
215009_s_at	THAP9-AS1	THAP9 antisense RNA 1	2,22E-06	−1,750
201108_s_at	THBS1	thrombospondin 1	1,50E-05	1,610
216997_x_at	TLE4	TLE family member 4, transcriptional corepressor	1,49E-05	−1,630
204427_s_at	TMED2	transmembrane p24 trafficking protein 2	5,61E-05	−1,680

ID	Symbol	Entrez Gene Name	Adj. *p*-Value	Log2 FC
218999_at	TMEM140	transmembrane protein 140	1,75E-05	1,490
217743_s_at	TMEM30A	transmembrane protein 30A	8,38E-07	−1,670
215275_at	TRAF3IP3	TRAF3 interacting protein 3	2,32E-06	−1,700
215047_at	TRIM58	tripartite motif containing 58	1,15E-05	1,850
201513_at	TSN	translin	9,50E-06	−1,460
209890_at	TSPAN5	tetraspanin 5	1,20E-07	1,450
201745_at	TWF1	twinfilin actin binding protein 1	3,34E-06	−1,460
212060_at	U2SURP	U2 snRNP associated SURP domain containing	1,62E-05	−1,400
206958_s_at	UPF3A	UPF3A regulator of nonsense mediated mRNA decay	4,59E-06	−1,450
201337_s_at	VAMP3	vesicle associated membrane protein 3	1,31E-05	−1,880
211571_s_at	VCAN	versican	1,24E-04	−1,630
215646_s_at	VCAN	versican	5,92E-04	−1,570
218940_at	VCPKMT	valosin containing protein lysine methyltransferase	6,37E-09	−1,880
210512_s_at	VEGFA	vascular endothelial growth factor A	3,07E-06	−1,620
206698_at	XK	X-linked Kx blood group	3,02E-04	1,650
220243_at	ZBTB44	zinc finger and BTB domain containing 44	1,77E-12	1,590
218645_at	ZNF277	zinc finger protein 277	3,67E-06	−1,510
